# Supplementary material for: Are carbon emissions trading and green financial instruments synergistic? -Comprehensive quantitative research based on content analysis
Source: PLoS One. 2024 Mar 7;19(3):e0298601. doi: 10.1371/journal.pone.0298601 (PMC10919601; doi:10.1371/journal.pone.0298601)
Supplement: S1 Data — (ZIP) [file pone.0298601.s001.zip › Data/地级市Z_F工作报告.zip!/║■─╧/╘└╤⌠╩╨/└┤╫╘╣1⁄2╓┌║┼í╛┬φ┐╦╩2╛▌═°í┐_2014.docx]

各位代表：

现在，我代表市人民政府，向大会报告政府工作，请予审议，并请市政协委员和其他列席人员提出意见。

2013年经济社会发展稳进向好

2013年，在中共岳阳市委的坚强领导下，在市人大、市政协的监督支持下，全市上下认真贯彻落实党中央、国务院和省委、省政府的决策部署，积极应对宏观经济下行压力，努力克服各种困难，顺利完成全年工作任务，在全面建成小康社会、建设美丽幸福岳阳的征程中迈出了坚实步伐。

1、经济总量持续增长。全年实现地区生产总值2430亿元，增长10.2%；完成财政总收入256亿元，增长11%。项目建设攻坚提速。高起点谋划和推进事关岳阳长远发展的重大战略，完成湖南长江经济带“1+7+1”规划体系、洞庭湖生态经济区“1+9”市域规划和中国洞庭湖博览园、南湖新城区概念性规划。湖南岳阳绿色化工产业园进入国家循环化改造示范试点园区，煤炭储备基地、百万千瓦火电厂、南翔万商国际商贸物流城、岳阳三荷机场等重大项目加快推进。实施项目建设“三个一批”竞赛活动和“重大项目建设百日攻坚行动”，百个重大建设项目97个开工，百个重大前期项目24个提前开工。完成固定资产投资1485亿元，增长27.2%。实体经济健康发展。深入开展帮扶企业行动，出台促进餐饮业发展十项措施、加快商贸流通业和现代物流业发展等政策文件，实体经济逐步企稳回升。规模工业增加值增长11.6%，石化、食品产业产值分别达1351亿元、999.7亿元。新增市场主体3.5万户，非公经济实现增加值1484亿元，增长12.3%，荣获全省非公经济考核第一名。生产要素保障有力。推进低丘缓坡未利用土地开发试点和存量建设用地再开发，保障了重大项目建设用地需求。完成7个110千伏以上变电站新建扩建和200个行政村配网改造工程。新增综合信贷118亿元，兴业银行岳阳分行、华容农村商业银行挂牌运营，新增5家小额贷款公司、4家保险公司、2家证券营业部，新增3家企业在天津股权交易所挂牌，成功发行18亿元国内首支“债贷组合”企业债，岳阳恒立复牌上市，胜景干黄在上海酒交所上市。举办各类招聘会、人才交流会300多场次，完成各类职业技能培训7.6万人次，引进外国专家122人次。农业生产总体平稳。奋力抗击严重干旱灾害，开展农村土地承包经营权确权登记颁证试点，新建高标准基本农田1.9万公顷，粮食总产310万吨，水产品总量稳居全省第一。湘阴、华容荣获全国粮食生产先进县。县域经济不断壮大。总体实力居全省第一方阵，汨罗进入全省县域经济强县前八强、首批特色县域经济重点县，湘阴、岳阳县进入县域经济发展先进县。经开区、汨罗、岳阳楼区财政总收入分别达21.6亿元、19亿元和18.3亿元，湘阴财政总收入突破10亿元。

2、发展质量加快提升。以“四化两型”、“三量齐升”为目标，着力转方式、调结构，三次产业结构调整为10.9∶55.1∶34。工业结构继续优化。电子信息、生物医药等新兴产业增加值增长15.3%，园区工业增加值增长12.3%，占规模工业比重分别提高到22.1%、42.5%，北斗导航产业基地建设和应用示范成效显著。节能减排深入推进。淘汰51条落后生产线，关闭43家污染严重企业，城区6家“退二进三”企业停产或转型，预计万元GDP能耗降低6%。完成污染减排项目121个，主要污染物排放削减率超额完成省定任务。科技创新步伐加快。全社会研发经费增长34.5%，实现发明专利授权量125件，高新技术产业增加值占GDP比重16.7%。获批创建岳阳国家农业科技园区，云溪精细化工产业基地成为省级创新型试点园区，临港产业新区启动创建国家高新区，临湘、平江荣获全国科技进步先进县市，岳阳楼区成为第二批国家智慧城市试点单位。城乡消费平稳增长。培育东茅岭、花板桥等城市商圈，推进“万村千乡”市场工程，完成社会消费品零售总额782亿元，增长13.8%。岳阳楼君山岛景区获评2013年度最受网民欢迎景区，“背诵《岳阳楼记》赠门票”活动社会反响良好；君山获评全省旅游产业发展十佳县市区，平江起义纪念馆、圣安寺、张谷英景区获评国家4A级旅游景区，湘阴文庙、左文襄公祠获批全国重点文物保护单位，旅游总收入增长20.7%。客运、货物周转量均增长14.9%，集装箱吞吐量突破20万标箱，港口货物吞吐量达1.1亿吨、居长江干线港口城市第六位。被《福布斯》中文版列为2013年中国大陆最佳商业城市。对外开放全面加力。推进区域合作，与咸宁、九江、安庆签订战略合作框架协议。积极参加“港洽周”、“中博会”、“湘商大会”等活动，成功举办第九届中国茶业经济年会暨首届中国（岳阳）黄茶文化节、岳阳（上海）旅游与重点产业项目招商推介会，华润、中储粮等14家国际国内500强企业落户岳阳，探索建立全省首个飞地工业园——长沙经开区汨罗产业园。内联引资到位资金316.5亿元，增长17.1%；实际利用外资2.8亿美元，增长21%；完成进出口总额6.4亿美元，增长11%。城陵矶口岸外贸联检锚地、进口肉类指定口岸查验平台、国际江海轮运输船队加快建设，综合保税区申报正式启动审核程序，长江航道整治工程和汽车整车进口口岸、粮食进口口岸等申报扎实推进，港澳水运直航实现常态运营。

3、惠民实事扎实办理。坚持抓发展与惠民生并重，人均均量稳步增长，民生支出占财政支出比重72%，新口径城乡居民收入分别达21193元和9930元。33项省市实事任务全面或超额完成，19项重大民生工程进展顺利，中心城区206条小街巷改造、5处低洼渍水地段整治和10856户农村危房改造全面完成。社会保障扩面提标。新增城镇就业5.6万人、农村劳动力转移就业6.3万人，城镇登记失业率3.96%。“五大保险”参保人数继续增加，企业退休人员养老金人均月增167.5元、达1448.9元；城乡低保人均月补差分别达270元和118元；新农合30种重大疾病政策范围内报销比例达80%以上。保障性住房和各类棚户区改造开工建设45476套。扶贫解困取得实效。8.8万农村贫困人口实现脱贫。平江在全省率先开展农村扶贫危房改造试点。出台《岳阳市实施<湖南省残疾人扶助办法>细则》，为3.5万名贫困重度残疾人发放特殊生活补贴金2000万元。市特困家庭大病医疗慈善救助基金达9600万元。社会事业繁荣发展。纪念建市30周年活动隆重节俭，评选表彰30位感动岳阳人物。李国武获评全国道德模范。组织高雅艺术演出15场、“欢乐潇湘·幸福岳阳”大型群众文艺汇演1253场，打造“周末一元剧场”品牌，花鼓戏《平民领袖》、《两份协议》分获第十届中国艺术节“文华剧目奖”和“群星奖”，获批第二批创建国家公共文化服务体系示范区。推进教育“阳光招生”，建成义务教育合格学校141所、农村公办幼儿园86所，成功举办首届校企联合发展大会。市一医院建成全省首批全科医生规范化临床培养基地，完成市二医院儿科综合病区和63家基层医疗卫生机构标准化建设项目。人口自然增长率控制在5.8‰以内，连续15年获评全省人口计生工作先进市，云溪、君山、华容、湘阴、汨罗、屈原获评全省模范单位。成功承办环中国国际公路自行车赛岳阳站比赛。

4、城乡环境明显改善。以“五创”提质为抓手，深入开展文明创建行动，城市文明程度指数测评排全国61个提名资格城市第五位，全国绿化模范城市正式授牌，国家园林城市顺利通过复查，荣获“全国无偿献血先进城市”称号，“文明餐桌行动”被央视《焦点访谈》推介，在全省城市卫生检查中排名第一。城市建设管理全面提质。洞庭新城、环南湖旅游交通三圈、南湖水上游乐、体育中心、档案馆等项目加快推进，王家河生态与文化长廊一期工程、中心城区亮化二期工程基本完成，公共自行车租赁系统投入运营。加强城市规划严控严管，启动城区“十大精品建筑”第三方评选活动。深入开展“六大整治”行动，整治规范城区市场36家、五小门店3600多家，改造排水窨井600多口，清理陈年垃圾2万多方，城市环境更加舒适宜居。全市城镇化率51%。基础设施建设加快推进。岳常、石华高速公路竣工通车，岳望、大岳高速和临湖公路等加快建设，建成干线公路134公里、农村公路332公里，完成危桥改造83座。竣工水利工程4.1万处，完成屈原、麻塘等堤垸堤防加固工程和9条中小河流治理、5个小农水重点县建设年度任务，整治病险水库256座，解决农村34.5万人饮水安全问题。完成造林30万亩。城乡面貌大为改观。强力推进禁拆治违，基本控住城区新增违法建设，拆除违法建设1611处21.7万平方米。加强铁山水资源管护，设立4000万元保护专项资金。持续开展“洞庭风雷”行动，推进河道采砂规范管理和湘江流域重金属污染治理，加大江豚救护力度，洞庭湖水环境和水资源得到有效保护。县城扩容提质成效显著，农村环境整治纵深推进，涌现一批秀美县城、秀美乡镇、秀美村庄和文明农户，华容、平江、岳阳县获评国家卫生县城。岳阳全年空气质量优良率88%，在全省6个重点监控城市中排名第一，被誉为“一座可以深呼吸的城市”。

5、民主法治不断健全。严格执行中央八项规定和省委、市委九项规定，切实加强民主法治和政府自身建设，政府执行力与公信力不断增强。依法治市深入推进。出台并实施《法治岳阳建设纲要》、《岳阳市人民政府工作规则》等，严格执行政府重大行政决策听证、规范性文件“三统一”和行政裁量权基准制度，认真落实市人大常委会决议决定和审议意见，自觉接受市人大依法监督和市政协民主监督，承办人大议案、代表建议和政协建议案、委员提案793件，落实率、满意率分别达98%和99.2%。政府职能加快转变。继续深化行政审批制度改革，精简审批环节40个，压缩审批时限三分之一以上；加强政府信息公开和诚信体系建设，健全完善政府采购、公共资源交易招投标等制度。在美国华盛顿中国研究中心发布的首个中国100大最佳市政府排行榜中，岳阳列第49位；在清华大学发布的中国市级政府财政透明度研究报告中，岳阳排全国285个地级城市第21位、居全省第一。

行政效能不断提升。深入开展“三提升”、“三项治理”、重大项目建设环境集中整治等主题活动，“市长信箱”荣获全国政府网站政民互动精品栏目奖，12345热线获评全国优秀热线。廉政建设扎实推进。认真落实国务院“约法三章”，合理编制“三公”经费支出预算，深入推进公务卡制度改革，全面停止新建楼堂馆所，基本完成办公用房清理，严格规范公职人员操办婚丧喜庆行为，强化公务用车监督管理，市本级“三公”经费支出同比下降10.6%。

6、社会大局和谐稳定。坚持把维护稳定作为第一责任，深入排查化解安全隐患和矛盾纠纷，社会更加安定有序。社会治理全面加强。积极开展“大走访”活动，推行“流动警务”，组织开展“三打一整治”等专项行动，加强网络舆情引导和应对，加强信访基层基础建设，信访形势总体平稳，连续7年获评全省社会管理综合治理先进市，君山排名全省第一，岳阳县、平江、岳阳楼区获评全省先进。安全形势持稳向好。扎实推进“打非治违”，持续开展安全生产大检查，突出危化品和水上安全整治，安全生产事故和死亡人数双下降，连续8年获评全省安全生产工作先进市，华容、汨罗、临湘获评全省先进。湘阴获评全国平安农机示范县。公共服务逐步完善。推进城市公交优先发展，投入1.06亿元，更新城区高档空调公交车200台。加快城市社区综合办公用房建设，推进社区居务公开，扩大农村社区建设试点。深入开展食品药品安全整治、打击假冒伪劣产品等专项行动，颁发首届市长质量奖。有效防控H7N9禽流感疫情，在全省率先实施动物疫病区域化管理。

其他各项事业协调发展，国防动员和后备力量建设取得新成绩，国安、人防、统计、史志、保密、工会、共青团、妇联、老干、民族宗教、社会科学、科协、气象、外事侨务、对台、工商联、驻外联络、住房公积金、移民、烟草、石油、邮政、通信、盐业等工作取得新进步。

这些成绩的取得，是省委、省政府和市委正确领导的结果，是市人大、市政协监督支持的结果，是全市人民团结奋斗的结果。在此，我代表市人民政府，向全市人民，向所有参与和关心支持岳阳建设发展的社会各界人士表示衷心的感谢！

各位代表，回顾一年工作，尽管我们作出了很大努力，经济社会发展“稳”的态势良好，主要经济指标均保持两位数增长，实现了保增长、保运转、保民生、保稳定的目标，但“进”的动力仍显不强，地区生产总值、规模工业增加值、财政总收入等主要指标增速趋缓，没有达到年初预期。究其原因，主要是：宏观经济趋紧的影响。去年全球经济增速放缓，全国经济进入高速增长向中高速增长的换挡期，全省及各市州经济增速下滑，普遍存在对宏观经济形势估计不足、主要经济指标预期偏高的问题。产业结构不优的影响。岳阳传统产业比重偏高，石化产业“一业独大”，其他优势产业规模偏小，巴陵、长炼两厂改扩建工程完成后，恰逢去年企业产量进入稳定期，新增量不大，加之化工产品市场需求和价格严重下降，因而对全市经济增长和财税增收的贡献减小。严重干旱灾害的影响。去年全市自然灾害频发，禽流感疫情对养殖业影响较大，特别是罕见的干旱灾害制约了农业增产、农民增收。工作推进还不平衡。各级各部门抓工作力度不一，实际效果参差不齐，少数地方在项目建设、产业发展、财税增收等方面差距较大，影响了全局。鉴于此，市政府本着实事求是的态度，向市人大常委会提交了《关于调整岳阳市2013年国民经济和社会发展目标部分指标的议案》，获得依法通过。同时，我们也清醒地看到，发展质量不优、城乡差距较大等问题仍然突出，民生改善、社会治理等任务仍然繁重，少数公职人员办事效率不高、服务态度不好、为政不廉等现象仍然存在。对此，我们将坚决采取措施，认真加以解决。

2014年加快全面建成小康社会进程

2014年是全面深化改革的开局之年，是完成“十二五”规划任务的攻坚之年，也是全面建成小康社会的关键之年。党的十八届三中全会吹响了全面深化改革的时代号角，省委、省政府作出了分类指导协调推进全面建成小康社会的战略部署，国家“两横三纵”城市化战略、长江中游城市群区域合作深入实施，长江开放经济带、洞庭湖生态经济区建设和产业梯度转移加快推进，必将为岳阳发展带来更多新机遇。同时，我们也面临经济发展整体趋缓、转型升级压力加大、深化改革任务艰巨、民生改善要求迫切、资源环境制约趋紧等诸多困难和挑战。全市上下一定要认清形势、坚定信心、抢抓机遇、奋力突破，确保岳阳在新一轮改革发展大潮中抢先发展，确保在全面建成小康社会进程中走在全省前列。

今年政府工作总体要求是：深入贯彻落实党的十八大及十八届二中、三中全会和中央、省委、市委经济工作会议精神，坚持“民本岳阳”一张蓝图干到底，以全面建成小康社会为总揽，把握稳中求进工作总基调，把改革创新贯穿于经济社会发展各领域各环节，转变发展方式，扩大对外开放，统筹城乡发展，创新社会治理，保障改善民生，推进“四化两型”，促进“三量齐升”，努力建设宜居宜业宜游的美丽幸福岳阳，努力让岳阳城乡更具现代气息、人民生活更加幸福美好。

今年全市经济社会发展主要预期目标是：地区生产总值增长10%；固定资产投资增长23%；规模工业增加值增长12%；社会消费品零售总额增长13%；财政总收入增长11%；城乡居民收入分别增长10%、12%；城镇登记失业率控制在4.1%以内；人口自然增长率控制在8‰以内；万元GDP能耗下降4%，全面完成省政府下达的主要污染物总量减排任务。

1、全面深化改革开放，增强发展内生动力

广泛凝聚改革共识，牢牢把握改革方向，全力推进改革，全面扩大开放，着力增强经济发展的动力与活力。

以更坚定的决心推进改革。加快政府职能转变，充分发挥市场在资源配置中的决定性作用，更好地发挥政府公共服务、市场监管、社会治理、环境保护的作用。深化行政审批制度改革，进一步简政放权，探索负面清单管理模式，推进工商注册制度便利化，加大政府购买公共服务力度。深化政府机构和乡镇站所改革。加快事业单位分类改革。落实财税体制改革，扩大“营改增”试点，推进全口径预决算、公务卡结算和预算绩效管理等改革，加强政府性债务管理，防范财政风险。完善金融市场体系，引进设立各类金融机构，加快农村信用社改革，发展多层次资本市场，强化金融监管，防范金融风险。处理好职工移交社区管理和电改、水改等市属国企改制遗留问题。建立透明规范的城市建设投融资机制，允许社会资本通过特许经营等方式参与城市基础设施投资和运营。推进市县政府性投融资平台改革，完善现代企业制度。深化农村综合配套改革，扩大农村土地承包经营权确权登记颁证试点，鼓励农村土地承包经营权依法流转，稳妥推进不动产统一登记和征地制度改革，探索建立城乡统一的建设用地市场。抓好水利五项改革，完成铁山灌区农业综合水价改革试点。加快户籍制度改革，促进农业转移人口市民化。积极推进教育、医药卫生、就业、收入分配等方面改革，建立更加公平可持续的社会保障制度。

以更开阔的视野扩大开放。充分发挥岳阳坐拥163公里长江黄金水道、地处湖南“一带一部”黄金节点和长株潭城市群与武汉城市圈黄金交点的区位优势，在扩大沿江开放、承接产业转移、加强区域合作等方面发挥“黄金作为”。积极发展对外贸易，继续实施外贸“百千亿”发展计划，促进进出口总额持续增长。加大招商引资力度，突出“长三角”、“珠三角”、港澳台等重点地区，瞄准国际国内500强企业和央省企业，着力引进一批大项目、好项目。鼓励寓外乡友返乡创业兴业。加快推进城陵矶进口肉类指定口岸建设，力争成功申报综合保税区、汽车整车进口口岸和粮食进口口岸，做强开放型经济发展平台。

以更高度的自觉推动创新。实施创新驱动发展战略，以科技创新促进产业转型升级，建设创新型岳阳。继续实施“312”工程，加快院士工作站、重点实验室、企业孵化器等创新平台建设，大力引进培育高端人才，建设人才强市。加快推进石油化工、装备制造、农业育种等重点领域技术攻关和成果转化，力争高新技术产业增加值占GDP比重达到20%。鼓励和支持企业开展信息化“登高计划”和“数字企业”创建行动，加大新产品、新工艺研发力度，提高企业自主创新能力，争创更多岳阳品牌。加强知识产权保护，营造鼓励创新、保护创新的良好氛围。

2、着力转变发展方式，推动经济转型升级

把经济工作重心转移到调结构、促转型上来，提高经济发展质量和效益，打造岳阳经济升级版。

大力推进新型工业化。加快湖南长江经济带建设，按照“四带一枢纽”的发展定位，加强港口岸线资源管理利用，做大做强绿色化工、电力能源、航运物流等产业集群，构建具有沿江特色的现代产业体系。立足优势产业和骨干企业，大力发展电子信息、先进装备制造、生物医药等战略性新兴产业，改造提升食品、造纸、纺织、建材、机电等传统产业，优化产业结构，提升产业素质。扎实推进洞庭湖生态经济区、“两型”社会滨湖示范区建设，深入开展节能减排与淘汰落后产能攻坚行动，加快实施“十大环保工程”，推行排污权交易制度，完成城区第一批、启动第二批“退二进三”工作，努力实现能耗和排放双下降。

加快发展现代农业。巩固农业基础地位，扎实推进环洞庭湖基本农田建设重大工程、小农水重点县和农田水利“百千万”工程建设，严格执行耕地保护和水资源管理制度，确保粮食生产稳定安全。加快水产品、生猪、茶叶、楠竹、油茶、蔬菜等优势产区建设，积极开展国家地理标志产品保护申报工作，扩大标准化、规模化种养，大力发展林下经济。强化农产品生产源头治理和产销全程监管，确保人民群众“舌尖上的安全”。健全完善农技推广服务体系，提高农业科技和机械化水平。大力扶持家庭农场、专业大户、农民合作社、产业化龙头企业等新型主体，支持农业电子商务平台建设。抓好以屈原为核心区的岳阳国家农业科技园区、汨罗特色县域经济重点县、平江国家扶贫开发工作重点县、君山全国休闲农业与乡村旅游示范区和屈原、华容国家现代农业示范区建设，支持岳阳县台湾农民创业园、君山黄茶产业园和洞庭湖绿色食品产业园建设，积极发展农产品加工园区和农副产品物流园区，继续实施“1351”龙头企业发展计划，提升农业产业化水平。

提速发展现代服务业。继续实施旅游产业“七个一”工程，加快推进中国洞庭湖博览园、屈子文化园、顺天洋沙湖国际旅游度假区等项目建设和休闲度假产业发展，积极推动洞庭湖旅游圈建设和高铁沿线旅游城市战略联盟，做强做旺“楼岛湖”和“花鸟舟情”品牌，打造令人流连忘返的旅游目的地。实施城市商圈提质工程，加快推进南翔万商国际商贸物流城和城市综合体、商务中心区建设，积极发展第三方物流，推动房地产业平稳健康发展，大力培育总部经济、楼宇经济、物流经济。引导商贸、餐饮等传统消费特色化、差异化发展，支持电子商务、服务外包、金融保险等生产性服务业发展，加快家政服务、健康养老、旅游休闲等生活性服务业发展，促进消费升温升级，推动服务业发展提速、比重提高、水平提升。

突出园区经济扩量提质。加快完善园区配套基础设施，推进标准化厂房建设，增强园区承载能力和服务功能。坚持特色兴园、主业强园，引导产业向园区聚集、企业向园区布局，提高园区产业集聚度和投入产出率。加快临港产业新区开发建设，积极创建国家高新区，打造全省综合交通运输体系的重要枢纽、现代物流的重要园区、开放型经济的重要门户。推动经开区在产业发展、城市管理、禁拆治违、队伍建设上突破求强，加快建设木里港工业区，规划好三荷空港产业区。推进湖南岳阳绿色化工产业园建设，延伸壮大丙烯、碳四、碳一、芳烃四条主产业链。加快汨罗“城市矿产”基地和飞地工业园建设。强化分类指导，支持各县市区优势产业优先发展，构建特色鲜明的园区经济体系，推动县域经济提质提速。

加力支持非公经济发展。毫不动摇鼓励、支持、引导非公经济健康快速发展，废除对非公经济各种形式的不合理规定，消除各种隐性壁垒，保证非公经济平等使用生产要素、公开公平公正参与市场竞争、同等受到法律保护。在全力服务央省企业持续扩张的基础上，更加注重培育壮大地方支柱产业和骨干企业，促进央省企业与地方企业良性互动发展。加大中小微企业扶持力度，强化政策引导，完善服务体系，提高中小企业发展质量，激发非公经济活力和创造力。加强非公企业监管和企业家培训，促进非公经济健康发展、非公经济人士健康成长。

3、狠抓重大项目建设，集聚更强发展能量

坚持以项目稳增长、调结构，继续开展重大项目引进和建设攻坚行动，重点推进 100个重大建设项目和 100个重大前期项目，力争年内完成投资 300亿元。

加快项目建设。抢抓长江开放经济带建设和老工业基地调整改造机遇，谋划推进煤炭储备基地、百万千瓦火电厂、城西老工业区搬迁改造、平益高速等重大项目建设，培育更多增长点。依托现有优势企业积极孵化项目，支持1500万吨炼化一体化、湖南科伦二期、远大可建三期等续建项目建设，推动企业扩张升级。坚持精干力量向项目集中，要素资源向项目集聚，加快推进泰格林纸70万吨涂布包装纸、北斗导航“两中心两基地”、中储粮油脂加工、胜景山河干黄产业园、华菱物流园等重大产业项目建设；加快推进蒙西华中铁路岳阳段、城陵矶新港二期、岳阳三荷机场、武警长沙（湘阴）直升机场、大岳和岳望高速、临湖公路、水库除险加固、电网升级改造等重大基础设施项目建设；加快推进中小河流治理、水土流失治理、马壕和罗家坡污水处理厂等重大生态环保项目建设；加快推进“三馆四中心”、农村饮水安全工程、市烈士陵园等重大民生实事项目建设。

提高项目质量。坚持招商选资、择优选项，把好项目规划关、环评关、效益关和质量关。优化项目规划布局，坚持以项目建设引领产业发展，实现同质集聚、错位发展。提高项目准入门槛，全面推行项目联合审批制度，坚决实行环评“一票否决制”，杜绝引进“三高一低”、工艺落后和产能过剩项目。依法制定项目用地和财税扶持政策，不断提高项目用地收益率和财税贡献率。严格工程质量和资金监管，确保项目施工安全和资金运行安全。

优化项目环境。实施项目审批流程再造，构建项目“绿色通道”，推动项目建设提速。加强项目资金保障，强化银企对接，鼓励和引导民间资本、保险资金参与重大项目建设，破除项目资金瓶颈。加大用地指标争取力度，大力开展闲置土地、违法用地清理整顿行动，用活用足低丘缓坡荒滩等土地试点政策，化解项目用地制约。继续开展帮扶企业和“三项治理”行动，努力营造亲商安商富商的浓厚氛围，让岳阳真正成为投资家的热土、创业者的乐园！

4、注重城乡统筹发展，促进城乡共同繁荣

坚持以城带乡、城乡互动，构建工农互惠、城乡一体的新型工农与城乡关系，加快全面建成小康社会步伐。

推进新型城镇化。坚持推进以人为核心的城镇化，提高城镇人口素质和居民生活质量，促进市区、县城和小城镇协调发展，促进城镇化和新农村建设协调推进。强化县乡规划引领与实施，加快县城路网、管网、广场、游园等配套设施建设，提升县城综合承载力。加快岳阳长江城镇带建设，促进临江与腹地联动、产业与城镇融合，构建分工有序、功能完备、布局合理的区域城镇体系。加快17个国家级重点镇创建工作，大力扶持20个省级特色镇和25个边界镇发展，建设一批园区型、边贸型、旅游休闲型等精品小城镇。继续抓好第三轮新农村建设10个城乡统筹试点镇、17个示范创建片、306个示范创建村建设，形成以点带面、整体推进的新农村建设格局。

建设水墨丹青岳阳。加快绿色岳阳建设，完善绿色环境、绿色产业、绿色消费、绿色文化体系，巩固绿化创模成果。认真落实湘江保护与治理省政府“一号重点工程”，推进临湘原桃林铅锌矿、平江尾砂库、岳阳县矾矿废渣、云溪松阳湖等区域重金属污染和农业面源、畜禽养殖污染综合治理。深入开展“洞庭风雷”行动，严厉打击非法采砂、捕捞、排污、营运等行为，保护洞庭湖生态环境和渔业资源。扩大城市绿色生态空间，加快洞庭湖风光带、城市公园、小游园建设，加强对金鹗山、尚书山、南湖等公园的生态保护和扩园提质，推进城区绿荫行动和公共场所立体绿化。切实加强城区水域保护，突出抓好南湖、东风湖水环境综合整治，严厉打击违法挖山、填湖、毁绿行为，把绿水青山留在城市、留给市民！加强东洞庭湖等自然保护区、铁山水库等重要水源涵养区和大云山、幕阜山、五尖山、福寿山、石牛寨等国家森林及地质公园保护，保障重要功能区生态安全。继续开展农村“四清五改”行动和秀美县城、秀美乡镇、秀美村庄、文明农户评选活动，推进农村环境整治常态化、长效化。加强PM2.5监控，公开发布全市空气污染指数，实施区域大气污染联防联控，加快淘汰黄标车，大幅削减主要污染负荷，让城乡居民共享清新空气、干净饮水和优良生态，让岳阳永远成为一座可以深呼吸的城市！

提高城乡公共服务水平。健全城乡发展一体化体制机制，推进城乡要素平等交换和公共资源均衡配置，逐步缩小城乡差距。完善城乡就业创业服务体系，支持屈原加快全省统筹城乡就业示范区创建步伐。稳步推进城镇基本公共服务常住人口全覆盖，把进城落户农民完全纳入城镇住房和社会保障体系。健全网络化城乡基层医疗卫生运行机制，促进优质医疗资源纵向流动。统筹城乡义务教育均衡发展，实行公办学校标准化建设，引导优质教育资源向农村学校、城镇办学困难学校辐射，保障流动人口子女随亲就读权利。加快完善城乡公共交通、文化体育、供水供电、邮电通信等基础设施，推进城乡最低生活保障制度统筹发展，促进城乡基本公共服务均等化。

5、深化文明创建行动，争创全国文明城市

加大文明创建力度，着力提高城市规划、建设和管理水平，争创全国文明城市。

完善城市功能。更加严格地执行城市总体规划和近期建设规划，加强重点区域、重点地段建筑立面景观设计，高标准完成城区防洪排涝、城市公共交通、岳阳大道（东）两侧城市设计等规划，继续开展城区“十大精品建筑”第三方评选活动，优化城市空间布局。实施城区扩容提质战略，加快临港产业新区、湖滨片区等路网建设，启动金凤湖生态保护区规划设计，支持洞庭新城、南湖新城区、云溪城南新区建设，推进跨湖发展，拓展城市发展空间。加快城市综合管沟、污水处理系统、公厕垃圾站、火车东站换乘中心和郭镇公交基地等站场建设，增强城市综合承载能力。

创新城市管理。深入开展城区“六大整治”、居民小区环境整治行动和爱国卫生运动，加快农集贸市场标准化建设和提质改造，突出抓好城市交通组织管理，完善道路微循环系统和公共自行车租赁系统，着力解决城区部分路段交通拥挤和停车难等问题，提高城市宜居指数。推进智慧城市建设，加快“三网融合”，大力发展物联网等新型业态和地理空间框架等应用系统，不断提升城市管理信息化水平。坚持疏堵结合，加大巡查禁违、依法拆违、从严治违力度，确保新增违法建设“零增长”、存量违法建设“负增长”，坚决查处搞违法建设的人和事，坚决打胜禁拆治违攻坚战、持久战。

彰显城市魅力。依托现有山水、文化等独特资源，促进城市与自然和谐共生、传统文化与现代文明交相辉映，努力建设有历史记忆和地域特色的魅力城市。突出滨水生态风格，继续推进“四湖两河”连通工程，加快王家河生态与文化长廊、环南湖旅游交通三圈建设，启动三大湖滨水休闲景观工程建设，着力打造水城岳阳、善水岳阳。坚持传承和弘扬优秀传统文化，加强名城、名镇、名村、名居保护，加快实施洞庭南路和陆城南北正街等历史街巷及建筑维修整治，延续城市历史文脉，增强城市文化底蕴。推进爱心城市建设，积极开展精神文明建设“六大行动”，弘扬主旋律，传播正能量，全面提高公民道德素质和社会文明程度，让岳阳这座城市充满爱心、充满感动！

6、积极创新社会治理，共建和谐幸福家园

把社会建设摆在与经济建设同等重要的位置，创新社会治理，维护公平正义，确保人民安居乐业、社会安定有序。

办好惠民实事。既量力而行又尽力而为，集中力量办好22件省市实事。继续实施城区“小街巷改造惠民三年行动计划”和“公交优先三年行动计划”，完成400条小街巷改造和200台高档空调公交车更新任务，科学规划设计公交专用道，提高城市公共服务水平；加快推进垃圾焚烧发电、基层粮库提质配套、农村30万人饮水安全等惠民工程，让人民群众得到更多实惠。加强价格监测预警，保持物价总水平基本稳定，保障困难群众基本生活。

健全保障体系。加快省级创业型城市建设，加大高校毕业生、城镇困难人员、失地农民等就业援助力度，保持零就业家庭动态清零，新增城镇就业5.2万人、农村劳动力转移就业3.2万人。推进社会保险扩面提质，不断提高基本社会保险覆盖率。加强住房保障体系建设，完成保障性住房建设和各类棚户区改造3.4万套，推进残疾人住房解困工作。完善社会救助体系，建成市残疾儿童康复中心、流浪未成年人救助保护中心，加大特殊困难群体帮扶力度，继续提高大病患者救助标准和城乡低保标准。实施精准扶贫，力争减少农村贫困人口8万人。完善养老服务体系，加快推进市社会福利院老年护理中心建设，鼓励和支持社会力量参与老年福利事业发展。

繁荣社会事业。坚持教育优先发展，依法保障教育投入，推进中小学债务清偿化解，建成53所义务教育合格学校、47所农村公办幼儿园，促进各类教育协调发展。推进文化惠民，继续实施文化强市十大工程，启动数字图书馆等公共文化设施建设，加快创建国家公共文化服务体系示范区。优化城区医疗资源布局，启动市中心医院建设，加快4家县级医院和55家乡镇卫生院建设，加强重大传染病防控，健全公共卫生服务体系。开展全民健身运动，办好省第八届少数民族传统体育运动会。坚持计划生育基本国策，促进人口长期均衡发展。搞好第三次全国经济普查和第一次全国地理国情普查。支持妇女儿童、工会、共青团、老干老龄等工作，促进广播电视、出版、档案、史志、社会科学、外事侨务等社会事业繁荣进步。

实现安全发展。毫不松懈抓好安全生产，提高安全生产考核权重，严格落实安全生产“一岗双责”制度，突出“打非治违”，加强危化品、水陆运输等重点领域和行业安全监管，坚决杜绝重特大安全事故发生。深入推进质量兴市战略。理顺食品药品监管体系，强化食品药品、农资和网购商品等市场监管，保障群众消费安全。完善突发事件应急管理和防灾减灾体系，提升应对突发事件综合处置能力。

确保社会稳定。加快平安岳阳建设，加强社会治安综合治理，深入开展“扫黑除恶”、打击“两抢一盗”、禁赌禁毒扫黄、打击非法集资、整治客运市场等专项行动，增强人民群众安全感。着力从源头上预防和减少社会矛盾，健全重大决策社会稳定风险评估机制，继续推动领导干部下访接访约访，加强网上信访办理，及时就地依法解决信访问题。加快法治岳阳建设，推进“六五”普法，完善人民调解、行政调解、司法调解联动工作机制。加快社区建设，创新流动人口、特殊人群服务管理，推进社会管理社区化、社区服务社会化。加强社会组织管理，激发社会组织活力。重视做好民族宗教工作，维护民族团结。加强虚拟社会管理，正确引导网络舆情。支持武警、消防部队能力建设，做好国防动员、国安、人民防空、征兵和双拥优抚工作，增进军政军民团结。

各位代表，全面完成今年各项工作任务，政府自身建设务必切实加强。坚持一心为民。践行为民服务的根本宗旨，凡是政府出台的政策、部署的工作，充分尊重群众意愿、凝聚群众力量、保障群众利益。扎实开展党的群众路线教育实践活动，坚决整治“四风”。强化底线思维和问题导向，坚持“功成不必在我”，多干打基础、利长远的实事，多办惠民生、得民心的好事。确保财政民生支出只增不减、民生实事办理力度只强不弱、城乡居民收入水平只升不降。坚持真抓实干。讲实话、办实事，不作假、不作秀，把更多的心思和精力用在谋发展、干事业上。发扬“钉钉子”精神，敢于担当，敢打硬仗，说了的坚决做到，定了的坚决执行，看准的事情一抓到底，部署的工作强力推进，善始善终、善做善成。以“三提升”活动为抓手，狠下决心治理“中梗阻”问题，着力提高行政效率、落实到位率和群众满意率。坚持依法行政。落实《湖南省行政程序规定》和《岳阳市人民政府工作规则》，健全法律顾问制度，完善重大行政决策听证、专家咨询论证和风险评估制度，确保政府及其部门依法高效运行。坚持党的核心领导，推进协商民主，主动接受市人大依法监督和市政协民主监督，认真落实市人大常委会决议决定和审议意见，扎实办理人大议案、代表建议和政协建议案、委员提案，虚心听取各民主党派、工商联、人民团体的意见，自觉接受社会公众监督，汇聚干事创业的强大合力。依法秉公办事，理性文明执法，确保行政权力在法治的轨道上运行，确保行政绩效经得起历史和实践的检验。坚持清廉清正。严格执行党风廉政建设责任制，坚持用制度管权管事管人，推进制度廉洁性评估工作和财政预决算、“三公”经费公开，加强对工程建设、土地出让、政府采购、公共资源交易等重点领域和重点环节的监管，强化行政监察和审计监督，把权力关进“笼子”里，把政务晒在阳光下。坚决落实中央八项规定和省委、市委九项规定，认真执行《党政机关厉行节约反对浪费条例》，艰苦奋斗，勤俭节约，全年“三公”经费和财政供养人员一律只减不增，政府性楼堂馆所一律不准新建，财政性资金建设项目超预算的一律不得擅自追加，市直单位富余办公用房一律统筹调剂利用，集中财力保民生、促发展。

各位代表，全面深化改革的征程已经开启，全面建成小康社会的目标催人奋进。让我们在中共岳阳市委的坚强领导下，凝聚全市人民的智慧和力量，铆足干劲，奋力拼搏，锐意进取，努力谱写建设美丽幸福岳阳的崭新篇章，努力让岳阳城乡更具现代气息、人民生活更加幸福美好！
